# Rapid and efficient high-performance liquid chromatography-ultraviolet determination of total amino acids in protein isolates by ultrasound-assisted acid hydrolysis

**DOI:** 10.1016/j.ultsonch.2024.107082

**Published:** 2024-09-25

**Authors:** Jorge A. Custodio-Mendoza, Patryk Pokorski, Havva Aktaş, Marcin A. Kurek

**Affiliations:** Department of Technique and Food Development, Institute of Human Nutrition Sciences, Warsaw University of Life Sciences (WULS-SGGW), 02-776 Warsaw, Poland

**Keywords:** Acid Hydrolysis, Fmoc-Cl, HPLC-UV, Plant-based Protein, Total AA, Ultrasound Assisted

## Abstract

This study presents the optimization and validation of an ultrasound-assisted acid method for the HPLC-UV determination of amino acids in plant-based proteins. The research focuses on enhancing hydrolysis efficiency and reducing environmental impact. Ultrasound treatment significantly accelerated hydrolysis by creating cavitation, which increases local pressure and temperature, leading to faster reaction rates. The optimal condition was a 30-minute treatment at 90 °C with 6 M hydrochloric acid. The 9-fluorenylmethyloxycarbonyl chloride derivatization was best performed at pH 9.0 using borate buffer, ethanol as the organic solvent, and a 5-minute derivatization time with a 5 mM concentration. The method’s analytical performance, validated according to FDA guidelines, showed excellent selectivity, specificity, linearity (r^2^ > 0.999), accuracy (recovery between 80–118 %), and precision (RSD<10.9). The analysis of 15 plant-based proteins revealed distinct amino acid profiles. Compared to traditional acid hydrolysis methods, the ultrasound-assisted approach demonstrated no significant difference in results (p-value > 0.05), confirming its reliability. The optimized ultrasound-assisted method is a reliable and efficient alternative for amino acid analysis, offering significant cost and time savings while maintaining high analytical performance. These findings are crucial for nutritional planning and developing functional foods to improve health outcomes.

## Graphical abstract

**Figure:**
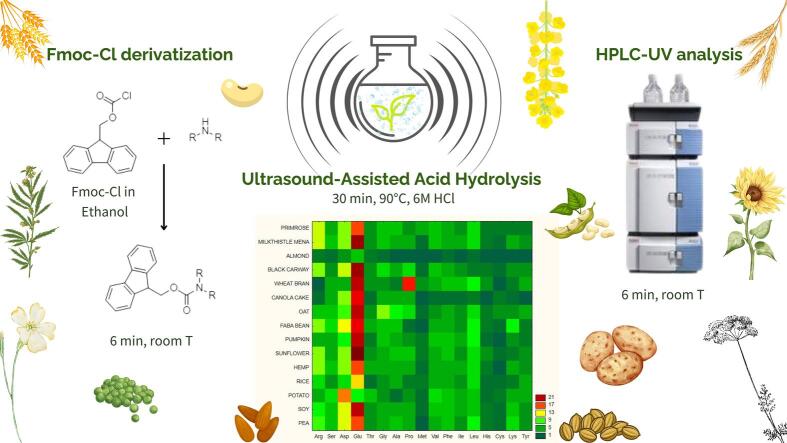

## 1 Introduction

Understanding protein isolates’ amino acid (AA) composition is essential because it directly influences their structure, functionality, and biological activity, impacting their potential health benefits and interactions in various applications [1], [2], [3]. Accurate AA profiling is critical for multiple applications, including nutritional planning, developing dietary supplements, and formulating functional foods to improve health outcomes [1], [2], [3].

Recently, there has been a growing interest in plant-based protein isolates due to their potential health benefits, environmental sustainability, and ethical considerations. These proteins offer a diverse range of AAs, meeting the nutritional needs of different populations, including vegetarians and vegans. The shift towards plant-based proteins aligns with global initiatives to reduce food production's environmental impact and promote sustainable agricultural practices [4], [5].

Accurately determining AA content in protein isolates is crucial for scientific research and industrial applications. This process typically involves hydrolyzing proteins into their constituent AAs, which are then derivatized for enhanced detection and quantification using chromatographic methods [6]. Hydrolysis is commonly performed using acid, base, or enzymatic methods. [6], [7], [8], [9]. Acid hydrolysis, often utilizing hydrochloric acid at 110 °C under nitrogen gas for 24 h, is a widely used technique [6], [10], [11], [12], [13], [14], [15], [16], [17]. However, it can degrade certain AAs, such as tryptophan, asparagine, and glutamine, leading to incomplete AA profiles and potentially compromising data accuracy [6]. To preserve and accurately quantify these three AAs, selective enzymatic hydrolysis is combined with alkaline hydrolysis using 4.2 M NaOH at 105 °C for 20 h [6], [7].

Conventional methods like reversed-phase high-performance liquid chromatography (HPLC) with fluorescence (FLD) or ultraviolet (UV) detection are commonly employed to analyze AAs in protein hydrolysates [3], [6], [7], [10], [12], [15]. Reverse-phase C18 columns are widely used for amino acid determination, especially after derivatization, which increases the hydrophobicity of the amino acids, enhancing their separation [3], [7], [10], [11], [15], [17]. Hydrophilic Interaction Liquid Chromatography (HILIC) columns have also been introduced, offering a complementary approach for separating highly polar amino acids [16]. Derivatization reagents such o-phthalaldehyde (OPA), 9-fluorenylmethyloxycarbonyl chloride (Fmoc-Cl), and 6-aminoquinolyl-N-hydroxysuccinimidyl carbamate (AQC) are used in pre-column derivatization to enhance the AA determination using HPLC and spectrophotometric detectors [10], [12], [15], [18]. AA reacts with OPA, forming highly fluorescent derivatives measured at excitation and emission wavelengths of 340 and 455 nm, respectively, or by UV at 340 nm [6], [15]. Fmoc-Cl derivates can be determined using either UV (at 262 nm) or FLD (excitation and emission wavelength of 254 and 313 nm, respectively) [10], [18]. AQC derivates can also be determined by UV (at 254 nm) and FLD (excitation and emission wavelength of 250 and 395 nm, respectively) [3], [12], [19]. While effective, these methods often involve prolonged reaction times and harsh conditions that can reduce efficiency and risk sample degradation [6]. Other approaches for the analysis of AA include capillary electrophoresis, gas chromatography-mass spectrometry (GC–MS), and liquid chromatography-tandem mass spectrometry (LC-MS/MS) [20], [21], [22].

Innovative approaches such as microwave-assisted acid hydrolysis have been developed to enhance accuracy and efficiency [3], [10], [12], [13]. Another promising technique is ultrasound-assisted hydrolysis, which leverages the power of ultrasound to improve hydrolysis efficiency [7], [8]. Ultrasound waves generate cavitation, leading to the rapid formation and collapse of solvent bubbles that increase local pressure and temperature, generating intense energy to break down molecular structures, including peptide bonds in proteins [23]. Additionally, ultrasound enhances mass transfer by creating microstreaming and turbulence, ensuring better contact between the sample and hydrolysis reagent, leading to faster and more complete AA release [23], [24]. Ultrasound has been used to enhance alkaline hydrolysis for determining amino acids in cosmetics by Du et al. and in human hair by Bhat et al. [7], [8].

Still, there is a need for accurate and precise methodologies for total amino acid determination in plant-based protein isolates that align with green chemistry principles, emphasizing the reduction of toxic solvents, energy consumption, and waste generation [25].

Here, we introduce a novel ultrasound-accelerated acid hydrolysis procedure for determining 17 amino acids in plant-based proteins using HPLC-UV. Our research demonstrates that ultrasound-assisted hydrolysis significantly reduces hydrolysis time while maintaining high analytical performance. By optimizing conditions such as treatment duration, temperature, and acid concentration, we achieved efficient hydrolysis in a fraction of the time required by traditional methods. The optimized method was validated according to the Food and Drug Administration guidelines, exhibiting excellent selectivity, specificity, linearity, accuracy, and precision.

This ultrasound-assisted acid hydrolysis procedure offers a reliable and efficient alternative for AA analysis in plant-based proteins, offering significant cost and time savings. These advancements are essential for functional foods’ nutritional assessment and development, contributing to better health outcomes and more sustainable food production practices.

## 2 Materials and methods

### 2.1 Chemicals & materials

Unless stated otherwise, all chemicals used in this study were of high purity (≥98 %). Sodium hydroxide, hydrochloric acid (35 %), phosphoric acid, disodium hydrogen phosphate, and sodium dihydrogen phosphate were purchased from Chempure (Germany). Formic acid (FA) and acetonitrile (ACN) were purchased from Poch (Poland). Methanol (MeOH) and ethanol (EtOH) were purchased from J.T. Baker (USA). Sodium tetraborate decahydrate was purchased from Honeywell, and sulfuric acid was purchased from Carl Roth. 9-Fluorenylmethoxycarbonyl chloride (Fmoc-Cl) was purchased from Merck (Darmstadt, Germany). Milli-Q water used in the experiments was prepared using a Millipore purification system in the laboratory.

To create standard solutions for analysis, analytical standards of AAs (AAS18) were also purchased from Merck, containing 2.5 mM L-Alanine (Ala), Ammonium chloride, L-Arginine (Arg), L-Aspartic acid (Asp), L-Glutamic acid (Glu), Glycine (Gly), L-Histidine (His), L-Isoleucine (Ile), L-Leucine (Leu), L-Lysine (Lys), L-Methionine (Met), L-Phenylalanine (Phe), L-Proline (Pro), L-Serine (Ser), L-Threonine (Thr), L-Tyrosine (Tyr), L-Valine (Val), and 1.25 L-Cystine (Cys) mM in 0.1 N HCl.

This study employed various laboratory equipment, including a Mettler Toledo (Spain) analytical balance (model ME104), a Hettich centrifuge (model 320/320 R), a ChemLand vortex mixer (model MX-S), a hotplate with magnetic stirring (model 06-HP550), a pH meter (model Basic 20) from Crison Instruments (Barcelona, Spain), a Binder drying oven (model FD115) from Merck, and an Elmasonic S 60H ultrasonic bath from Elma Ultrasonics (Germany) to conduct all experiments.

### 2.2 Sample acquisition, purification, & quality control for total AAs determination

15 plant-based protein powder isolates were purchased from local stores in Warsaw, Poland. These included peas, Soy, Potato, Rice, Hemp, Sunflower, Pumpkin, Faba bean, Oat, Canola cake, Wheat bran, Black caraway, Almond, Milk thistle, and Primrose. All samples were stored in their original packaging at room temperature and protected from light until the study

If the protein purity was below 80 %, proteins were purified according to the method described by Aydemir et al. (2022) [26]. Briefly, 10 g of the sample was diluted in 150 mL of ultrapure water, and the pH of the solution was adjusted to 10 using 1 M NaOH to ensure complete dissolution of the protein. The mixture was stirred at room temperature for 1 h. The solution was centrifuged at 9000 rpm for 20 min, and the supernatant was collected. The pH of the solution was adjusted with 1 M HCl to reach the isoelectric point of each protein (data not shown) to induce protein precipitation. The solution was centrifuged again, and the precipitated protein was redissolved in water. The pH was adjusted to 7.0 to avoid further affecting the extraction procedure, and the sample was freeze-dried overnight.

Quality control samples (QCs) were freshly prepared by adding standard solutions to a rice protein isolate at 10, 30, and 60 μg/mL concentrations. These QCs were stored at −20 °C for no more than one week to maintain their integrity and consistency for subsequent analytical assessments.

### 2.3 Ultrasound-assisted acid hydrolysis and derivatization of plant-based proteins

**Fig. 1:**
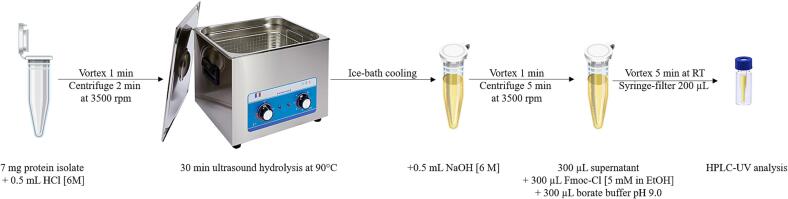
Scheme of Ultrasound-Assisted Acid Hydrolysis with Fmoc-Cl Derivatization for HPLC-UV Determination of Amino Acids in Plant-Based Proteins.

The process of ultrasound-assisted acid hydrolysis and subsequent derivatization of a plant-based protein isolate for total AA determination is outlined in Fig. 1. Briefly, 7 mg of the protein isolate was accurately weighed and transferred into a 15 mL conical bottom tube. Subsequently, 0.5 mL of a 6 M HCl solution was added to the tube, vortexed at room temperature for 1 min to homogenize, and centrifuged for 2 min at 3500 rpm to pellet residual sample material from the tube walls into the solution. The tube was then placed into a preheated ultrasound bath set at 90 °C and incubated for 30 min to accelerate hydrolysis. After the incubation period, the tube was carefully removed from the ultrasound bath, cooled using an ice bath, and 0.5 mL of a 6 M NaOH solution was added to stop the hydrolysis reaction. The tube contents were then vortexed for 1 min and centrifuged at 6000 rpm for 5 min to separate the phases. Following centrifugation, 0.3 mL of the upper phase was collected using a pipette and transferred to a clean tube. To derivatize the released AAs, 0.3 mL of borate buffer (pH 9.0) and 0.3 mL of 5 mM Fmoc-Cl reagent were added to the tube. The contents were then vortexed for 5 min at room temperature to ensure complete derivatization. The mixture was filtered through a 0.25 µm PTFE filter, and the filtrate was transferred into a chromatographic vial fitted with a 200 µL insert for HPLC-UV determination.

#### 2.3.1 Traditional acid hydrolysis

To assess the new method's performance, a comparison was made with a traditional acid hydrolysis approach using a protocol based on Jaudzems & Fuerer (2022) [27]. Initially, 7 mg of protein isolate was placed in a 6 mL headspace vial and treated with 0.5 mL of 6 M HCl. The mixture was vortexed for 1 min and then heated in an oven at 120 °C for 24 h to hydrolyze the proteins. Subsequently, the tube was cooled using an ice-water bath, and 0.5 mL of 6 M NaOH was added to stop the reaction and neutralize the solution pH. After further vortexing and centrifugation at 4500 rpm for 5 min, the upper layer containing the total AAs was collected for derivatization. Derivatization was performed as described above.

### 2.4 High-performance liquid chromatography-ultraviolet analysis

LC-UV analysis was performed using a ThermoFisher Scientific system, which included an Accela Autosampler (60057–60020), a quaternary pump (600), and a photodiode array (PDA) detector operating at 40 Hz with a 5 cm LightPipe™ flow cell. Analyte separation was achieved using a 2.6 µm Kinetex C18 column (2.1 × 50 mm) from Phenomenex (CA, USA). The mobile phases consisted of 0.1 % formic acid (A) and ACN (B) in a gradient mode, starting at 75 % A and decreasing to 50 % A over 13.23 min. The composition was further decreased to 25 % A until 14.33 min, returned to the initial conditions by 15.44 min, and held for 4.66 min. The injection volume was 10 µL. The total analysis time was 20.1 min. The PDA detector simultaneously scanned from 200-600 nm to obtain the full UV spectrum of analytes and monitored wavelengths at 250, 260, and 270 nm.

#### 2.4.1 MS/MS analysis

To identify each Fmoc-AA derivate, a tandem mass spectrometry (MS/MS) experiment was conducted using a similar setup, coupling the HPLC with a Thermo Scientific LCQ Fleet™ Ion Trap LC/MSn system. Separation was done using the Kinetex C18 column and the mobile phase program described before (section 2.4). The ESI probe operated at a capillary temperature of 350 °C and voltage of 38 V, using nitrogen for sheath and auxiliary functions and high-purity helium for collision-induced dissociation at 35 % energy. Mass spectrum was monitored from 50 to 1000 *m*/*z* in multi-reaction monitoring (MRM) mode.

### 2.5 Experimental design & statistical analysis

An asymmetric factorial 2^3^3^1^ design was employed to investigate the influential factors of the methodology across eight experiments. This design choice reduced the required runs compared to a full factorial design, which would have required 12 experiments. The factors under screening included buffer type (phosphate and borate), Organic solvent (ACN and EtOH), derivatization time (5 and 10 min), and Fmoc-Cl concentration (2, 5 and 10 mM). The impact of the methodology was described using the model in equation (1).

(1) y=b0+∑i=16biBAxBA+biCBxCB+biCAxCA

In this equation, A, B, and C correspond to levels 1, 2, and 3 for the different factor coefficients (b_i_). This model characterizes how substituting one level of a factor for another affects the US-accelerated acid hydrolysis method, with “y” representing the peak area obtained in the HPLC-UV analysis. For example, coefficient b1/21 defines the impact of replacing the buffer phosphate level with borate, b_2/12_ describes the effect of replacing a solvent ACN with EtOH, and so on. The constant term b_0_ represents the mean response from all experiments. NemrodW® statistical software was used to generate the experimental design, assess the data, and visualize its effects.

### 2.6 Analytical validation & method performance assessment

A rice protein isolate sample was used as the blank sample for analytical validation of the method, adhering to guidelines from the Food and Drug Administration (FDA) for the Validation of Chemical Methods in Food, Feed, Cosmetics, and Veterinary Products, and the Bioanalytical Method Validation Guidance for Industry [28], [29].

Method specificity was established by selecting a maximum UV wavelength for all analytes, each eluting at distinct retention times. The absence of interference in these elution regions confirmed method specificity. Standard addition calibration curves were built from the lower limit of quantification (LLOQ) to the upper limit of quantification (ULOQ) for each analyte. These curves were used to determine the in-sample LOD and LLOQ using equation (2).

(2) Y_(LOD/LLOQ)=Y_blank+(3.3/10)σ_blank

LLOQ and ULOQ denote each analyte's lowest and highest concentrations, which can be quantitatively determined with acceptable precision and accuracy. Standard addition calibration curves were built at six concentration levels to assess method linearity. QCs at 10, 30, and 60 μg/mL were used to evaluate method accuracy through recovery (n = 3) and precision via intraday and interday assays, expressed as %RSD (n = 5). Moreover, QCS were also used to construct Shewhart Individuals Control Charts to monitor daily sequence acceptability during sample analysis. Individual measurements from quality control samples were plotted to build these charts, with the central line representing the average of these measurements. The upper and lower control limits were calculated as the average plus three times the standard deviations of the QCs measurements. Similarly, the upper and lower warming limits were calculated as the average plus two times the standard deviations.

## 3 Results and discussion

### 3.1 O ptimization of ultrasound-assisted acid hydrolysis and derivatization of plant-based proteins

Selecting a proper blank sample is crucial in developing an analytical method to serve as a control. In this work, we used a rice protein isolate as a blank sample based on the AOAC Food Matrix Triangle [28], categorizing protein materials by their protein, fat, and carbohydrate content. Plant-based protein isolates, including rice protein, contain over 67 % protein and less than 33 % fat and carbohydrate content. Therefore, all of them can be analyzed under the same category. This choice ensured that the blank sample effectively represented the baseline composition and matrix effects relevant to all plant protein isolates studied.

**Fig. 2:**
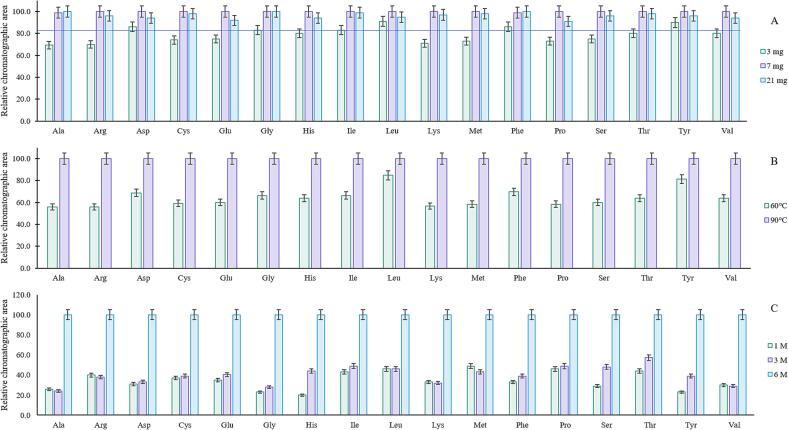
Study of the effect of sample size (A), temperature (B), and acid concentration (C) on the effectiveness of protein hydrolysis for total amino acids determination. L-Alanine (Ala), L-Arginine (Arg), L-Aspartic acid (Asp), L-Cystine (Cys), L-Glutamic acid (Glu), Glycine (Gly), L-Histidine (His), L-Isoleucine (Ile), L-Leucine (Leu), L-Lysine (Lys), L-Methionine (Met), L-Phenylalanine (Phe), L-Proline (Pro), L-Serine (Ser), L-Threonine (Thr), L-Tyrosine (Tyr), L-Valine (Val).

Various parameters affecting the method's performance were assessed. First, we aimed to reduce the sample size as much as possible without compromising the method's sensitivity. As shown in Fig. 2A, a similar response in terms of relative chromatographic area (comparative peak percentages) is achieved when using 7 mg and 21 mg protein isolate. Although for Asp, Leu, Phe, and Tyr, a 3 mg sample is also similar to larger sample size signals, the relative chromatographic area decreases by 80 % for the rest of the AAs. Thus, 7 mg protein isolate was selected for further experiments, which also allows the use of a reduced acid volume.

Ultrasound enhances hydrolysis by cavitation, creating intense local pressure and temperature fluctuations in the reaction system, increasing the reaction rate and efficiency. The relationship with temperature is significant because the heat generated by ultrasound can further accelerate hydrolysis, improving the breakdown of proteins. However, excessive temperatures can degrade sensitive compounds, so optimal temperature control is crucial for effective hydrolysis. The effectiveness of a 1-hour ultrasound treatment at 60 °C and 90 °C was explored (Fig. 2B), resulting in a higher relative chromatographic area when using 90 °C. Since higher temperatures were not explored due to instrumental limitations, 90 °C was set for further experiments. Furthermore, Fig. 2C shows a remarkable difference when using different HCl concentrations. For instance, 1 or 3 M HCl barely reaches up to 40 % relative chromatographic area compared to 6 M HCl. Thus, 6 M was selected for further experiments.

Sulfuric acid and o-phosphoric acid (pKa 2.1) were studied as alternatives to hydrochloric acid since they can reach low pH in solution with a lower environmental impact (Supplementary Material, Fig. 1SA). Results in terms of chromatographic area showed that for ala, cys, his, ile, leu, pro, ser, tyr, and val, sulfuric acid reached almost similar areas to HCl but lower areas for the rest of the AAs, as did o-phosphoric acid, which reached significantly lower results. Given the need for a strong enough signal for quantification and the use of a reduced acid volume, it was decided to continue using HCl. Further, the volume of acid was studied at 1 mL and 0.5 mL, having bigger chromatographic signals with the smaller volume. Lower than 0.5 mL was not possible due to incomplete dilution of the protein isolate.

**Fig. 3:**
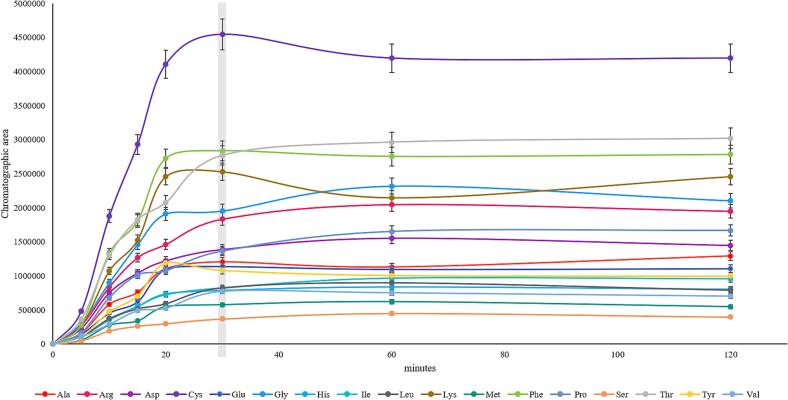
Kinetic study of the ultrasound-accelerated acid hydrolysis for total amino acids determination. L-Alanine (Ala), L-Arginine (Arg), L-Aspartic acid (Asp), L-Cystine (Cys), L-Glutamic acid (Glu), Glycine (Gly), L-Histidine (His), L-Isoleucine (Ile), L-Leucine (Leu), L-Lysine (Lys), L-Methionine (Met), L-Phenylalanine (Phe), L-Proline (Pro), L-Serine (Ser), L-Threonine (Thr), L-Tyrosine (Tyr), L-Valine (Val).

A kinetic study of the ultrasound-assisted acid hydrolysis with 0.5 mL HCl [6 M] at 90 °C was performed (Fig. 3). Although after 20 min, ala, ser, val, tyr, lys, and phe were already completely released, 30 min ensured the complete release of all AAs from the protein isolate. On the other hand, Fmoc-Cl derivatization, which required mild alkaline conditions, was tested at pH 8.0 and 9.0 using borate buffer. Fig. 1S.C shows that the best results are achieved using pH 9.0.

**Fig. 4:**
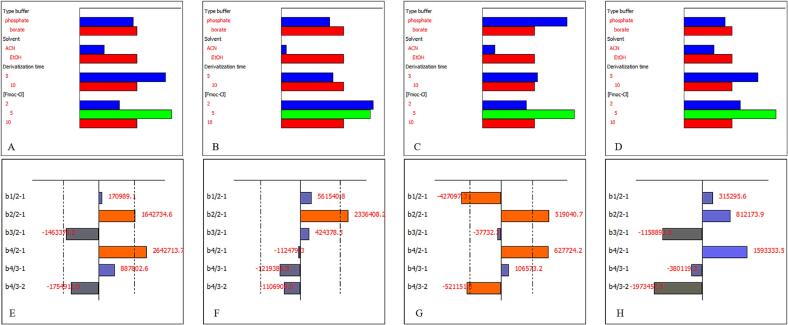
Total Effect and Delta Weight charts for L-Aspartic acid (A, E); L-Lysine (B, F); L-Serine (C, G); and L-Valine (D, H).

Moreover, the results of the asymmetrical factorial design on the effectiveness of buffer type, organic solvent, derivatization time, and derivatization reagent concentration in the ultrasound-assisted acid hydrolysis and derivatization of plant-based proteins revealed several key findings. The total effect and delta weight chart indicated that using borate buffer yielded better results, though this improvement was not statistically significant. Ethanol as the organic solvent positively affected the method's effectiveness and was statistically significant for most AAs. While a 10-minute derivatization time was optimal for lysine, a 5-minute derivatization time significantly improved the method's effectiveness for the remaining AAs. For the concentration of the derivatization reagent, 5 mM Fmoc-Cl showed the longest bar for most AAs, indicating its effectiveness, while 10 mM Fmoc-Cl had a negative statistical effect. Based on these findings, the optimal conditions were borate buffer, ethanol as the organic solvent, a 5-minute derivatization time, and a 5 mM concentration of Fmoc-Cl (Fig. 4).

Our Ultrasound-Assisted Acid Hydrolysis for HPLC-UV determination of total amino acids in plant-based protein isolates procedure distinguishes itself by significantly reducing the hydrolysis time and temperature conditions, the usage of HCl, and replacing the use of ACN by EtOH compared to prior determination of acid hydrolysis methods for total AAs in protein isolates [11], [14], [16], [17]. To our knowledge, no analytical methods had been developed to use ultrasound for protein hydrolysis acceleration.

### 3.2 Analytical features of the ultrasound-assisted acid hydrolysis procedure

**Fig. 5:**
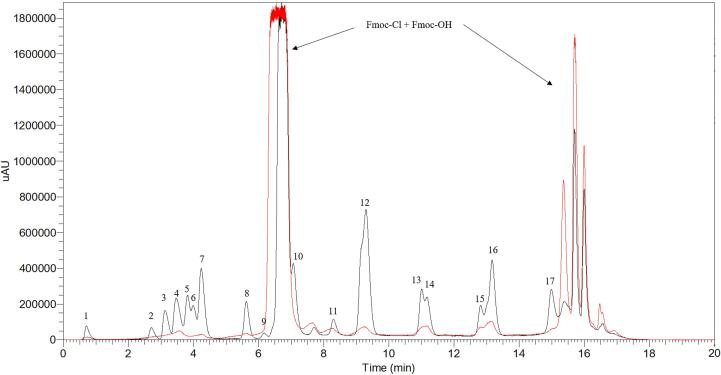
LC-UV Chromatogram of 1 µg/mL Amino Acid Standards (black) vs Derivatization Solution Blank (red): 1. L-Arginine, 2. L-Serine, 3. L-Aspartic acid, 4. L-Glutamic acid, 5. L-Threonine, 6. Glycine, 7. L-Alanine, 8. L-Proline, 9. L-Methionine, 10. L-Valine, 11. L-Phenylalanine, 12. L-Isoleucine, 13. L-Leucine, 14. L-Histidine, 15. L-Cystine, 16. L-Lysine, 17. L-Tyrosine. μAU stands for micro-Absorbance Units.

Following FDA guidelines [28], [29], we thoroughly evaluated the analytical performance of the Ultrasound-Assisted Acid Hydrolysis for HPLC-UV determination of total AAs after the Fmoc-Cl derivatization procedure. We began by assessing selectivity and specificity, identifying the maximum UV absorbance with specific retention times for the Fmoc derivates of Arg, Ser, Asp, Glu, Thr, Gly, Ala, Pro, Met, Val, Phe, Iso, Leu, His, Cys, Lys, and Tyr. All AAs showed distinct retention times and specific spectra (Table 1), with no matrix interferences observed in the HPLC-UV chromatogram (Fig. 5). MS/MS data were used for confirmation of the Fmoc-AA derivates (Table 1S).

**Table 1:** Analytical figures of merit for ultrasound-assisted acid hydrolysis method in HPLC-UV determination of amino acids in plant-based proteins.

Amino acid	RT	UV max	LOD	LOQ	Calibration curves			Accuracy			Intra-day precision			Inter-day precision		
								% recovery			% RSD			% RSD		
	min	nm	µg/g	µg/g	m	b	r^2^	L-QC	M−QC	H-QC	L-QC	M−QC	H-QC	L-QC	M−QC	H-QC
Arg	0.71	272	0.15	0.50	71.99	32,496	0.9996	96.4	112.5	95.6	2.6	4.6	5.3	10.8	10.2	7.0
Ser	2.70	262	0.15	0.50	28.84	47,368	0.9994	104.8	104.2	90.3	8.8	5.5	1.3	6.8	2.4	7.4
Asp	3.13	262	0.08	0.25	67.61	54,794	0.9994	110.8	118.8	115.3	5.2	6.6	5.5	4.2	10.7	2.9
Glu	3.47	262	0.08	0.25	36.67	35,929	0.9994	116.0	80.6	106.0	4.4	9.7	9.6	4.7	2.9	1.7
Thr	3.38	262	0.10	0.33	103.3	125,169	0.9993	105.5	106.7	107.7	3.4	7.5	9.0	1.1	4.4	10.3
Gly	3.98	262	0.30	1.00	178.7	140,847	0.9999	106.5	96.7	92.2	1.7	9.7	9.3	10.9	7.3	10.4
Ala	4.25	262	0.04	0.14	170.8	160,370	0.9991	104.0	118.9	88.5	3.2	3.2	9.7	4.9	2.7	5.3
Pro	5.62	262	0.06	0.20	139.2	123,612	0.9996	108.7	95.5	116.7	6.6	4.1	9.6	10.7	8.7	6.2
Met	6.16	258	0.30	1.00	83.38	52,771	0.9997	106.4	115.6	91.8	8.3	9.4	9.9	6.2	5.8	9.8
Val	7.07	262	0.08	0.25	62.00	38,946	0.9998	114.1	108.8	114.9	5.7	3.5	8.6	4.8	4.7	2.6
Phe	8.27	253	0.15	0.50	21.61	96,092	0.9992	113.6	80.9	92.3	3.2	8.6	5.6	9.6	2.5	9.9
Ile	9.29	262	0.02	0.06	47.52	121,897	0.9999	98.6	87.9	93.7	5.4	7.1	7.2	6.7	6.4	1.2
Leu	10.98	261	0.08	0.25	46.12	177,747	0.9997	98.2	96.7	110.9	6.7	6.3	4.6	3.8	3.7	4.9
His	11.16	261	0.15	0.50	107.6	121,511	0.9997	116.7	116.9	80.9	9.3	8.2	9.8	6.9	10.8	6.7
Cys	12.79	259	0.10	0.33	25.95	17,050	0.9991	91.4	90.7	85.2	8.2	5.9	9.4	4.2	5.7	6.9
Lys	13.14	262	0.04	0.14	21.12	77,573	0.9997	103.8	80.4	116.3	2.7	7.2	2.3	4.0	8.2	6.9
Tyr	14.99	250	0.06	0.20	28.77	28,211	0.9992	113.4	110.9	99.7	2.5	2.8	4.3	4.0	2.9	2.4

L-QC, low-level quality control substance at 10 μg/m; M−QC, middle-level quality control substance at 30 μg/mL; H-QC, high-level quality control substance at 60 μg/mL; RSD, relative standard deviation.

The method's characteristics, including detection limits, linearity, accuracy, and precision, are summarized in Table 1. Standard addition calibration plots covered a concentration range from the lower limit of quantification (LOQ) (0.06–1 µg/mL) to 100 µg/mL, demonstrating excellent linearity (r2 ≥ 0.9991). Accuracy evaluation using QC samples showed recoveries ranging from 80.4 % to 118.9 %, while both intraday and interday precision yielded RSD values ≤ 9.9 % and ≤ 10.9 %, respectively, meeting predefined acceptability criteria (Table 2).

**Table 2:** Amino acid content of plant-based proteins (part 1).

	Pea		Soy		Potato		Rice		Hemp		Sunflower		Pumpkin		Faba bean	
	mean	± SD	mean	± SD	mean	± SD	mean	± SD	mean	± SD	mean	± SD	mean	± SD	mean	± SD
Arg	8.41	0.02	7.81	0.03	4.46	0.01	6.33	0.01	9.94	0.06	9.75	0.05	4.62	0.01	9.80	0.03
Ser	5.42	0.02	5.22	0.04	6.04	0.03	3.91	0.01	5.21	0.02	4.52	0.03	4.46	0.01	6.43	0.01
Asp	11.94	0.06	11.82	0.08	15.46	0.02	6.93	0.02	9.43	0.05	9.78	0.06	7.16	0.02	12.47	0.08
Glu	16.56	0.01	20.64	0.09	7.77	0.03	13.97	0.08	16.16	0.07	25.47	0.14	20.71	0.15	18.72	0.01
Thr	3.82	0.03	3.93	0.01	5.58	0.02	1.17	0.01	4.56	0.01	3.71	0.01	2.19	0.01	4.70	0.02
Gly	4.01	0.03	4.42	0.03	3.45	0.02	3.52	0.01	4.02	0.01	4.92	0.01	5.17	0.01	5.94	0.03
Ala	5.42	0.03	4.51	0.03	1.34	0.01	4.47	0.03	4.50	0.01	5.01	0.02	4.85	0.04	5.32	0.03
Pro	4.41	0.02	4.91	0.01	3.31	0.01	2.81	0.02	4.52	0.01	5.14	0.01	3.82	0.01	1.60	0.01
Met	0.91	0.01	1.40	0.01	1.62	0.01	2.28	0.01	1.40	0.01	2.02	0.01	2.58	0.01	0.20	0.01
Val	4.91	0.02	5.11	0.04	6.23	0.01	4.51	0.02	4.99	0.02	4.50	0.01	5.61	0.03	4.21	0.02
Phe	5.75	0.02	5.50	0.01	5.46	0.03	4.42	0.01	4.57	0.01	5.59	0.01	5.34	0.02	4.73	0.02
Ile	3.90	0.01	4.20	0.01	4.37	0.01	3.47	0.01	4.00	0.01	3.81	0.02	4.15	0.03	3.93	0.01
Leu	7.64	0.05	5.62	0.04	5.82	0.02	6.46	0.01	6.65	0.03	6.92	0.04	7.89	0.01	7.82	0.06
His	2.40	0.01	2.51	0.01	1.30	0.01	1.81	0.01	2.83	0.01	3.09	0.01	1.53	0.01	2.80	0.01
Cys	1.00	0.01	1.21	0.01	2.44	0.01	1.61	0.01	0.17	0.01	1.47	0.01	0.45	0.01	1.01	0.01
Lys	6.70	0.03	5.62	0.01	4.35	0.01	2.40	0.02	4.17	0.01	2.91	0.02	3.41	0.01	7.74	0.04
Tyr	3.99	0.01	3.89	0.01	4.04	0.01	4.22	0.01	3.70	0.01	3.19	0.01	2.89	0.01	2.61	0.02

Results expressed in mg/100 mg of protein isolate. SD, standard deviation

Table 2S presents a comparison of chromatographic methods for AA determination previously published. These methods reported instrumental determination limits similar to or higher than those presented here [7], [10], [11], [14], [15], [21]. Notably, the use of high-resolution mass spectrometry led to significantly lower determination limits, as reported by Kambhampati et al. in the determination of 20 AAs in soy flour [16], Danielsen et al. in the determination of 18 AAs in Lucerne [17], and Zhou et al. in the determination of 21 free AAs in tea [22]. HRMS allows sensitive determination of AAs without derivatization, achieving similar determination limits to those presented here [14] or lower [13]. Determination coefficients reported are at least 0.999, similar to those reported by Liyanaarachchi et al. [21]. In contrast, previously published methods with r2 values below 0.999 explain less of the data's variance, indicating less precise fits and weaker predictive power [3], [7], [10], [11], [12], [13], [14], [15], [16], [17], [22]. Our method demonstrates recovery values closer to 100 % than previously published methods, highlighting its remarkable accuracy and reliability. Specifically, our method achieves near-perfect recoveries in determining 20 AAs in pollen and soy flour. Moreover, the precision of our method aligns with previously reported methods [11], [12], [13], [14], though we observe lower relative standard deviations when using MS or FLD detectors [7], [14], [17], [21]. Most methods have been developed for a sole protein source [7], [10], [11], [13], [14], [15], [16], [17], [21], [22]. Regarding the reported analytical features, our method shows superior performance and robustness compared to those developed for various protein sources [3], [12].

### 3.3 AA profiling of plant-based proteins using ultrasound-assisted acid hydrolysis

**Fig. 6:**
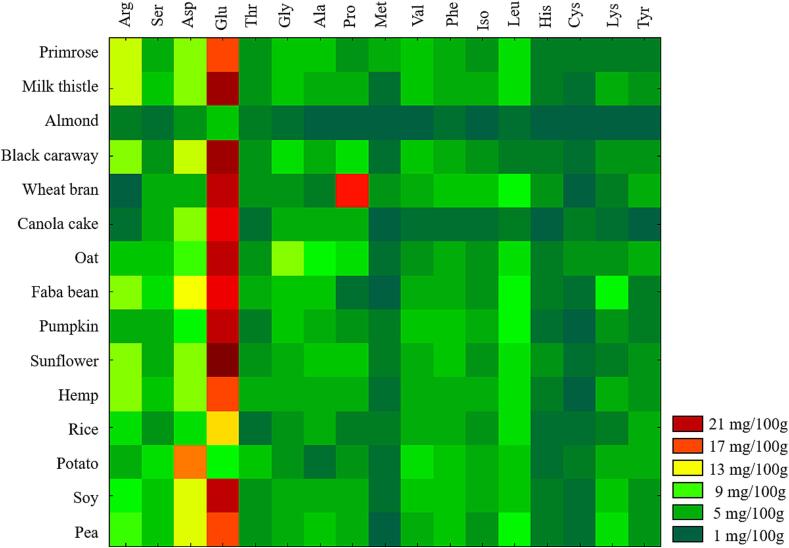
Heatmap of Total Amino Acid Concentration in Plant-Based Protein Isolates.

The AA content of 15 plant-based proteins was determined using the validated ultrasound-assisted acid hydrolysis method. The sample analysis results were considered valid only when the measurements of the QCs fell between the control limits of the Shewhart Individuals Control Chart (Fig. 2S). Results expressed as mg/100 mg of protein (Fig. 6 and Table 2, Table 3) reveal significant variability in the AA content across different protein sources. Notably, Sunflower, Wheat Bran, Black Caraway, and Milk Thistle exhibit exceptionally high levels of Glutamic Acid, essential for protein synthesis and AA metabolism. Potato has the highest Aspartic Acid content, vital for energy production and nutrient metabolism. Arginine levels are notably elevated in Hemp, Milk Thistle, and Primrose, indicating potential cardiovascular and immune benefits. Essential AAs such as Lysine and Leucine are abundant in pea, Faba Bean, and pumpkin, which are crucial for tissue repair and muscle function. Although low across most sources, methionine is highest in Primrose, emphasizing the need for dietary variety or supplementation in plant-based diets. These results underscore the importance of combining different plant proteins to achieve a balanced amino acid (AA) intake, which is particularly crucial for vegetarians and vegans. Additionally, these findings highlight the potential for targeted use of specific plant proteins in functional foods to enhance health outcomes, such as improving cardiovascular health and aiding in muscle repair.

**Table 3:** Amino acid content of plant-based proteins (part 2).

	Oat		Canola cake		Wheat bran		Black Caraway		Almond		Milk thistle		Primrose	
	mean	± SD	mean	± SD	mean	± SD	mean	± SD	mean	± SD	mean	± SD	mean	± SD
Arg	5.51	0.02	1.89	0.01	0.30	0.01	9.17	0.02	2.37	0.01	10.10	0.02	10.21	0.05
Ser	5.80	0.01	4.33	0.02	4.68	0.01	3.80	0.01	1.34	0.01	5.36	0.03	4.33	0.04
Asp	8.21	0.01	9.04	0.03	4.18	0.01	10.08	0.03	3.90	0.01	9.76	0.02	9.05	0.07
Glu	20.45	0.07	18.55	0.14	20.98	0.01	22.46	0.17	5.72	0.01	22.62	0.05	16.11	0.02
Thr	3.92	0.01	1.34	0.01	3.09	0.02	3.96	0.04	2.71	0.01	3.91	0.02	3.58	0.01
Gly	9.16	0.02	4.19	0.01	3.43	0.01	6.86	0.03	1.49	0.01	5.93	0.03	5.10	0.04
Ala	7.23	0.03	4.30	0.01	2.75	0.02	4.23	0.01	0.90	0.01	4.28	0.02	5.57	0.04
Pro	6.34	0.02	4.72	0.01	17.58	0.02	6.10	0.02	0.90	0.01	4.25	0.01	3.33	0.02
Met	1.00	0.01	0.58	0.01	3.89	0.02	1.45	0.01	0.19	0.01	1.24	0.01	4.60	0.01
Val	3.21	0.01	1.64	0.01	4.40	0.01	5.12	0.01	0.94	0.01	5.19	0.01	5.14	0.01
Phe	4.97	0.02	1.31	0.01	5.35	0.03	4.01	0.01	1.17	0.01	4.14	0.01	4.10	0.01
Ile	3.55	0.01	1.27	0.01	5.31	0.01	3.98	0.01	0.83	0.01	4.18	0.01	3.36	0.02
Leu	6.88	0.01	2.23	0.01	7.27	0.05	2.84	0.01	1.52	0.01	6.81	0.01	6.76	0.03
His	2.14	0.02	0.81	0.01	3.98	0.02	2.84	0.01	0.51	0.01	2.52	0.01	2.83	0.02
Cys	3.30	0.01	2.21	0.01	0.79	0.01	1.18	0.01	0.27	0.01	1.60	0.01	2.53	0.01
Lys	3.46	0.01	1.79	0.01	2.36	0.01	3.94	0.01	0.62	0.01	4.47	0.03	2.79	0.01
Tyr	4.32	0.03	0.39	0.01	4.29	0.01	3.35	0.02	0.65	0.01	3.54	0.02	2.63	0.01

Results expressed in mg/100 mg of protein isolate. SD, standard deviation

When comparing the results of AA content obtained using the ultrasound-assisted acid hydrolysis method with those obtained through the traditional acid hydrolysis method, there is no statistical difference, as indicated by the p-value and the Bland-Altman plots (Fig. 3S). The Bland-Altman plot provided a visual representation of the differences between the two methods for each amino acid, allowing for an assessment of how closely they agree. The results indicated that most amino acid differences lay between the limits of agreement, suggesting that the two methods provide comparable results. However, aspartic acid (Asp) and glutamic acid (Glu) were exceptions, as their differences were slightly out of the limits of agreement or right on the lines.

Moreover, the AA content reported here is consistent with those reported in previous studies by various authors [30], [31], [32], [33], [34], [35], [36], [37], [38], [39], [40], [41]. Specifically, Tang et al. documented a comparable AA profile in hemp protein isolate to the findings presented here [30]. Similarly, Gorissen et al. identified analogous AA content in oat, hemp, soy, pea, and potato proteins, aligning with our results [31]. Oikama et al. reported a similar AA composition in potato protein isolates, and Liu et al. found consistent values for soy and pea protein isolates [32], [33]. Kalman’s rice protein AA content findings also match those reported here [34]. Additionally, House et al. observed a similar AA profile in almond protein [35]. Li et al. reported consistent AA content in wheat bran, while Vinayashree et al. found similar profiling in pumpkin proteins [36], [37]. Hadidi et al. reported comparable AA content in primrose, and Vioque et al. noted similar AA profiling in faba protein [38], [39]. Lastly, Grageola et al. reported AA content in canola cake that aligns with our findings, and Petraru et al. observed similar AA content in sunflower protein [40], [41]. This consistency across various research findings reinforces the reliability and validity of the method presented. It indicates that the ultrasound-assisted acid hydrolysis method is a robust alternative to the traditional method, providing comparable results.

## 4 Conclusions

The optimization of ultrasound-assisted acid hydrolysis and derivatization for plant-based proteins emphasizes the importance of selecting an appropriate blank sample. A rice protein isolate was used, reflecting the composition and matrix effects pertinent to all plant protein isolates studied. Critical parameters, such as reducing sample size and optimizing hydrolysis, were optimized to enhance the method's performance. Ultrasound was shown to improve hydrolysis efficiency by increasing reaction rates through cavitation. The optimal conditions were determined to be a 30-minute treatment at 90 °C using 6 M HCl. Although alternatives like sulfuric and o-phosphoric acid were considered, HCl proved most effective.

Furthermore, the Fmoc-Cl derivatization required mild alkaline conditions, with pH 9.0 providing the best results. An asymmetrical factorial design highlighted borate buffer, ethanol as the solvent, a 5-minute derivatization time, and an optimal 5 mM concentration of Fmoc-Cl. The method notably reduced hydrolysis time and temperature, improved efficiency, and replaced acetonitrile with ethanol, making it a robust procedure for HPLC-UV determination of total AAs in plant-based protein isolates.

Analytical performance was rigorously evaluated following FDA guidelines, demonstrating excellent selectivity, specificity, linearity, accuracy, and precision. The method achieved high recoveries and low relative standard deviations, showing superior performance and robustness compared to existing methods. Additionally, the method was validated through comparison with traditional acid hydrolysis, showing no significant difference, thus confirming its reliability.

The AA profiling of 15 plant-based proteins revealed significant variability, underscoring the importance of dietary diversity for vegetarians and vegans. Strategic combinations of different plant proteins can ensure a balanced AA intake, improving health outcomes. The consistent results with previous studies validate the ultrasound-assisted method as a reliable alternative for AA analysis in plant-based proteins, which is crucial for accurate nutritional planning and research.

## CRediT authorship contribution statement

**Jorge A. Custodio-Mendoza:** Writing – original draft, Visualization, Validation, Investigation, Formal analysis, Conceptualization. **Patryk Pokorski:** Visualization, Investigation, Formal analysis. **Havva Aktaş:** Investigation. **Marcin A. Kurek:** Writing – review & editing, Supervision, Project administration, Conceptualization.

## Declaration of competing interest

The authors declare that they have no known competing financial interests or personal relationships that could have appeared to influence the work reported in this paper.
