# Supplementary material for: Rapid and efficient high-performance liquid chromatography-ultraviolet determination of total amino acids in protein isolates by ultrasound-assisted acid hydrolysis
Source: Ultrason Sonochem. 2024 Sep 25;111:107082. doi: 10.1016/j.ultsonch.2024.107082 (PMC11470170; doi:10.1016/j.ultsonch.2024.107082)
Supplement: Supplementary Data 1 [file mmc1.docx]

A

B

C

**Figure 1S**. Impact of Acid Type (A) and Volume (B), and Derivatization pH (C) on the Effectiveness of Ultrasound-Accelerated Acid Hydrolysis with Fmoc-Cl Derivatization. L-Alanine (Ala), L-Arginine (Arg), L-Aspartic acid (Asp), L-Cystine (Cys), L-Glutamic acid (Glu), Glycine (Gly), L-Histidine (His), L-Isoleucine (Ile), L-Leucine (Leu), L-Lysine (Lys), L-Methionine (Met), L-Phenylalanine (Phe), L-Proline (Pro), L-Serine (Ser), L-Threonine (Thr), L-Tyrosine (Tyr), L-Valine (Val).

Table 1S. MS/MS Data of Fmoc-Cl Derivatized Amino Acids

| **Amino Acid** | **Q1** | **Q3** | **Collision Energy** |
| --- | --- | --- | --- |
|  | **Precursor Ion** | **Product Ion** |  |
|  | **m/z** | **m/z** | **v** |
| L-Arginine | 397 | 134 | 12 |
| L-Serine | 328 | 130 | 11 |
| L-Aspartic acid | 378 | 263 | 20 |
| L-Glutamic acid | 370 | 179 | 14 |
| L-Threonine | 342 | 120 | 10 |
| L-Glycine | 320 | 263 | 8 |
| L-Alanine | 334 | 263 | 20 |
| L-Proline | 360 | 115 | 13 |
| L-Methionine | 371 | 174 | 12 |
| L-Valine | 340 | 118 | 21 |
| L-Phenylalanine | 410 | 263 | 11 |
| L-Isoleucine | 354 | 130 | 11 |
| L-Leucine | 354 | 131 | 20 |
| L-Histidine | 377 | 154 | 20 |
| L-Cystine | 344 | 147 | 21 |
| L-Lysine | 591 | 179 | 17 |
| L-Tyrosine | 404 | 181 | 9 |

Table 2S. Comparative Analysis of Amino Acids Determination by Chromatographic Methods: Analytical Features and Performance

| Sample | Amino acids  n | Hydrolysis | Derivative reagent | Instrument | LOQ  μg/g | r^2^ | Accuracy  % Recovery | Precision  % RSD | Reference |
| --- | --- | --- | --- | --- | --- | --- | --- | --- | --- |
| Pollen | 20 | MAAH | Fmoc-Cl | HPLC-FLD | 0.05-1.0 | > 0.998 | 98-101 | < 3.8 | 10 |
| Microalgae | 23 | AH | MTBSTFA | GC-MS | 0.2 | > 0.990 | 70-120 | < 23.89 | 11 |
| Infant milk, Egg Yolk, Beef extract, Cattle bone, Collagen, Peanut, Corn | 16 | MAAH | AQC | HPLC-PDA | 0.75 | > 0.990 | 72-135 | < 25.3 | 12 |
| Yeast | 16 | MAAH | * | UHPLC-QTOF | 0.7-14 x10^-3^ | > 0.980 | 64-98 | < 36 | 13 |
| Lentils and BSA | 20 | AH | * | HPLC-HRMS | 0.0001-0.25 | > 0.997 | 70-87 | < 55.4 | 14 |
| Microalgae | 19 | PAH | OPA | HPLC-DAD | 0.3-1.4 | > 0.992 | NR | NR | 15 |
| Soy flour | 20 | AH | AQC | LC-HILIC-HRMS | 2-6 x10^-6^ | NR | 86-99 | NR | 16 |
| Lucerne | 18 | AH | S-NIFE | LC-MS/MS | 0.012-0.062 | > 0.990 | 65-95 | < 5.2 | 17 |
| Cosmetics | 7 | USBH | EACA | HPLC-FLD | 0.29-0.5 | > 0.993 | 79-114 | < 3.7 | 7 |
| Quinoa, amaranth, buckwheat | 17 | MAAH | AQC | UHPLC-PDA | 20-130 | > 0.997 | NR | NR | 3 |
| Rice | 16 | ** | * | HPLC-MS/MS | 1-8 | > 0.999 | 80-110 | < 7.0 | 21 |
| Tea | 21 | ** | AQC | UHPLC-MS/MS | 0.5-105 x^-6^ | > 0.994 | 81-108 | < 14.1 | 22 |
| Pea, Soy, Potato, Rice, Hemp, Sunflower, Pumpkin, Faba bean, Oat, Canola cake, Wheat bran, Black caraway, Almond, Milk thistle, Primrose | 17 | USAH | Fmoc-Cl | HPLC-PDA | 0.14-1.0 | > 0.999 | 80-118 | < 10.9 | This work |

N, number of amino acid determined; LOQ, limit of quantification; r^2^, determination coefficient; RSD, relative standard deviation; MAAH, Microwave Assisted Acid Hydrolysis; Fmoc-Cl, 9-fluorenylmethoxycarbonyl chloride; HPLC, High-Performance Liquid Chromatography; FLD, Fluorescence Detector; AH, acid hydrolysis; MTBSTFA, N-Methyl-N-(tert-butyldimethylsilyl)trifluoroacetamide; GC-MS, Gas Chromatography – Mass Spectrometry; AQC, 6-aminoquinolyl-N-hydroxysuccinimidyl carbamate ; PDA, Photo Diode Array detector; UHPLC, Ultra-High-Performance Liquid Chromatography; QTOF, Quadrupole Time-of-Flight detector; HRMS, High Resolution Mass Spectrometry; PHA, pressurized acid hydrolysis; OPA, o-Phthaldialdehyde; DAD, Diode Array Detector; HILIC, Hydrophilic Interaction Liquid Chromatography; S-NIFE, (S)-N-(4-nitrophenoxycarbonyl) phenylalanine methoxyethyl este; MS/MS, Tandem Mass Spectrometry; USBH, Ultrasound Assisted Basic Hydrolysis; EACA, ε-aminocaproic acid; USAH, Ultrasound-Assisted Acid Hydrolysis; NR, not reported.

* underivatized; ** free amino acids


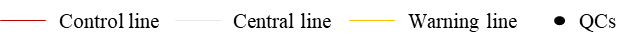


**Figure 2S**. Shewhart Individuals Control Charts.


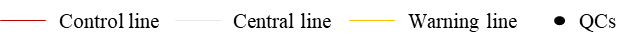


**Figure 2S**. Continue.


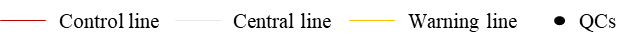


**Figure 2S**. Continue.


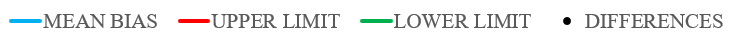


**Figure 3S**. Bland-Altman Plot Comparing Amino Acid Content from Traditional and Ultrasound-Assisted Acid Hydrolysis Methods.


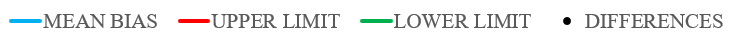


**Figure 3S**. Continue.


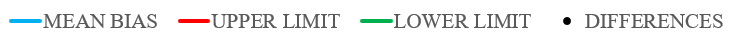


**Figure 3S**. Continue.
